# Association Between Children’s Theory of Mind and Responses to Insincere Praise Following Failure

**DOI:** 10.3389/fpsyg.2018.01684

**Published:** 2018-10-10

**Authors:** Ai Mizokawa

**Affiliations:** Graduate School of Education and Human Development, Nagoya University, Nagoya, Japan

**Keywords:** theory of mind, insincere praise, performance rating, motivation, young children, Japanese

## Abstract

This study examined children’s interpretations of and responses to insincere praise in a situation involving failure and explored the association between these responses and the maturity of their theory of mind. Seventy-two young Japanese children (mean age = 5.70 years, *SD* = 0.61) completed a test battery that included tasks designed to assess responses to teacher feedback (i.e., insincere praise, no feedback) in hypothetical failure situations, theory of mind, and verbal ability. The results showed that children who failed experienced higher levels of positive emotion and self-rated performance and showed lower motivation to persevere when they received insincere praise following failure, relative to those observed when they failed and received no feedback. In addition, relative to children with less mature theory of mind, children with mature theory of mind responded more negatively to insincere praise following failure. The evidence indicated that the effects of insincere praise could differ depending on the maturity of children’s theory of mind. It highlights the importance of understanding individual differences in theory of mind in parenting and educational settings.

## Introduction

Children are exposed to diverse types of evaluative feedback regarding their behavior, intelligence, and performance in their social lives. Parents and teachers often provide children with positive feedback to enhance their intrinsic motivation and boost their self-esteem (Henderlong and Lepper, 2002; Brummelman et al., 2014). However, an increasing body of evidence suggests that praise is not always beneficial for children (see Dweck, 2000; Henderlong and Lepper, 2002 for a review). For example, unlike process-related feedback (e.g., “you worked hard”), receiving personal feedback (e.g., “you are smart”) following success, when children’s positive performance is attributed to their personal traits, could foster resistance to subsequent mistakes (Mueller and Dweck, 1998; Kamins and Dweck, 1999).

Notably, Henderlong and Lepper (2002) suggested that the effects of praise on motivation depend on not only the praise content (e.g., process-related feedback or personal feedback) but also the recipients’ characteristics and interpretation of the praise. Evidence accumulated from research conducted thus far has revealed that some variables, such as perceived autonomy and performance standards, affect children’s motivation (e.g., Deci et al., 1999; see Henderlong and Lepper, 2002 for a review). However, few studies have examined insincerity, another key variable, or have assessed the manner in which children interpret and respond to praise that leaves room for doubt. Developmental studies have shown that young children can produce and understand insincere utterances from around the age of 4 or 5 years (Airenti and Angeleri, 2011; Bosco and Gabbatore, 2017). Although previous literature has not directly examined the sensitivity of children to insincerity in praise, this evidence leads us to speculate that children around the age of four or five come to notice insincerity in praise that is given in an inappropriate situation (i.e., failure situation), and begin to display negative responses to insincere praise.

The question as to how and when children should be praised is one of the main concerns of adults engaged in parenting and education; however, very few attempts have been made to examine the impact of praise in situations where children fail and evaluate their own performance as poor. Indeed, evaluations received from others are not always congruent with children’s own self-evaluation; for example, parents and teachers sometimes praise children when the children themselves feel that they did not perform well on a task (i.e., when they have failed). A recent study that examined children’s responses to inflated praise found that school-aged children with low self-esteem sought fewer challenges after they received inflated praise (Brummelman et al., 2014). Crucially, this finding indicated that children with low self-esteem might evaluate their own performance as poor compared to children with high self-esteem, and the disagreement between internal low evaluation and external high evaluation (i.e., inflated praise) might cause the negative response to the praise, because they might recognize the praise as insincere.

This study also focused on children’s theory of mind that could be one of the key factors that serve to constrain their interpretation of and responses to insincere praise. Theory of mind is defined as the ability to attribute mental states to the self and others to interpret behavior (Premack and Woodruff, 1978). False-belief tasks are commonly used to measure young children’s theory of mind development, and a large body of evidence has shown that children pass first-order false-belief tasks between the ages of 3 and 5 years (Wellman et al., 2001). Individual differences in theory of mind in early childhood influence children’s social lives in diverse ways (Hughes, 2011). Theory of mind allows children to comprehend non-literal speech, such as irony (e.g., Happé, 1993), white lies (Broomfield et al., 2002), and antisocial lies (Talwar and Lee, 2008), by understanding the intention behind the words that are spoken. That is, as theory of mind develops, children can infer the intention underlying non-literal speech and detect its true meaning. Children need to interpret the meaning of praise and compliments in situations where they receive insincere praise. Therefore, in situations where children are praised following failure, they may use theory of mind to determine the reason for the praise.

Ambiguity in praise, which is characteristic of feedback in Japanese culture, is another key topic that has been overlooked in previous research. The Japanese language is highly contextualized, and the content of the speaker’s discourse is ambiguous, rather than explicit (Minami, 2002). In Japan, ambiguity is observed not only in language but also in parenting and education. Japanese mothers adopt an “absorbent childrearing” approach, where they do not teach children explicitly but encourage them to understand situations and behave autonomously (Azuma, 1994). In addition, Japanese preschool and kindergarten educators use the *machi no hoiku* (childrearing of waiting) strategy, whereby they hold back and wait, allowing children the opportunity to handle problems on their own with minimal assistance from teachers or adults (Tobin et al., 2009; Hayashi and Tobin, 2014), and this strategy might support both positive and negative self-reflection. Against a cultural background involving this language system and these educational beliefs, Japanese children might be more likely to receive ambiguous evaluative feedback without an apparent reason (e.g., “great,” “good,” or “okay”) relative to Western children.

To my knowledge, Kayo (2002) conducted the first study to examine ambiguous praise, where the experimenter suddenly approached 2- to 4-year-old children while they were engaged in free play at preschool and provided them with praise with no indication of the reason for which praise had been provided (“great!” or “*sugoi!*” in Japanese). Children received this feedback in situations where they had not performed activities that was appropriate for evaluation (e.g., success, failure), and their facial and verbal reactions were observed. The results showed that most 2-year-old children and some 3-year-old children smiled and showed agreement with the sudden ambiguous praise, while some 3-year-old children and most 4-year-old children exhibited confusion at the sudden ambiguous praise, with dubiety in their facial expressions, and half of them asked about the reason for the praise. These findings suggest that at approximately 4 years of age, children begin to consider not only praise itself but also the reason for praise. Although Kayo (2002) observation indicated that 4-year-old children were confused by ambiguous praise and cared about the reason for which praise had been offered, children’s responses to and interpretation of ambiguous praise in situations involving activities that evaluation is appropriate for (e.g., success, failure) remained unclear.

The overall aim of the current study was to examine young Japanese children’s interpretations of and responses to insincere praise, which involves ambiguity in the reason of the praise, and explore the association between these responses and the maturity of their theory of mind. The first hypothesis was that participants would display a negative emotional response, low performance rating, and low motivation to insincere praise, similar to that observed in the failure situation without feedback, since they notice that the praise is insincere. The second hypothesis was that most participants would attribute the receipt of insincere praise following failure to their efforts rather than outcomes or their personal attributes. This hypothesis was proposed based upon the Japanese cultural belief system, which emphasizes the importance of hard work, effort, and persistence in self-improvement (e.g., Tuss et al., 1995; Heine et al., 1999). Their tendency to attribute the insincere praise to their efforts may be the basis for low motivation after receiving insincere praise, as this includes the message that “they had already made a sufficient effort.” The third hypothesis concerned the relationship between theory of mind and the child’s responses to insincere praise. It was hypothesized that children with mature theory of mind would be more likely to report negative responses (i.e., negative emotion, low levels of self-rated performance, and weaker motivation for perseverance) after receiving insincere praise following failure, relative to children with less mature theory of mind. The proposed reason for this was that, whereas children without mature theory of mind could consider the praise as sincere if they take it at face value, children who possess mature theory of mind could interpret the hidden message behind the praise and consider it invalid. Therefore, when children possess mature theory of mind, their positive emotion, performance rating, and motivation for further challenge could be reduced because they speculate the reason of the praise in relation to the failure situation.

## Methods

### Participants

Seventy-two Japanese children aged 4 years and 7 months to 6 years and 9 months participated (mean age = 5.70 years, *SD* = 0.61; 24 boys, 48 girls). All participants were native Japanese speakers attending one of two preschools. An ethics approval for psychological research was not required at the time the research was planned and conducted as per the author’s institution guidelines (Meiji Gakuin University) as well as national regulations. Though there was no opportunity to submit an ethics application at that time, the author carried out this study in accordance with the recommendations of the Japanese Psychological Association Ethical Principles of Psychologists with careful attention to ethical considerations to uphold high ethical standards. All participants’ parents gave the author written informed consent in accordance with the Declaration of Helsinki. Although detailed demographic information regarding the children could not be obtained because of school privacy policies, the sample was recruited predominantly from middle-class families.

### Materials and Procedure

The children were tested individually in quiet rooms at the preschools. They performed a failure-praise task, theory of mind tasks, and a verbal ability test. The order of the tasks was counterbalanced.

#### Failure-Praise Task

A new task designed to measure individual differences in responses to praise following failure was developed for this study. Children’s responses were examined by using stories in which they appear as a protagonist puppet; then, they were asked how they felt in the stories. The task structure and procedure were based on sensitivity-to-criticism tasks that have been used to assess children’s responses to criticism in both Eastern and Western countries (e.g., Cutting and Dunn, 2002; Lecce et al., 2011, 2014; Mizokawa, 2013, 2015; Mizokawa and Lecce, 2017). The stories that were used in the tasks are shown in **Appendix 1**.

**FIGURE 1:**
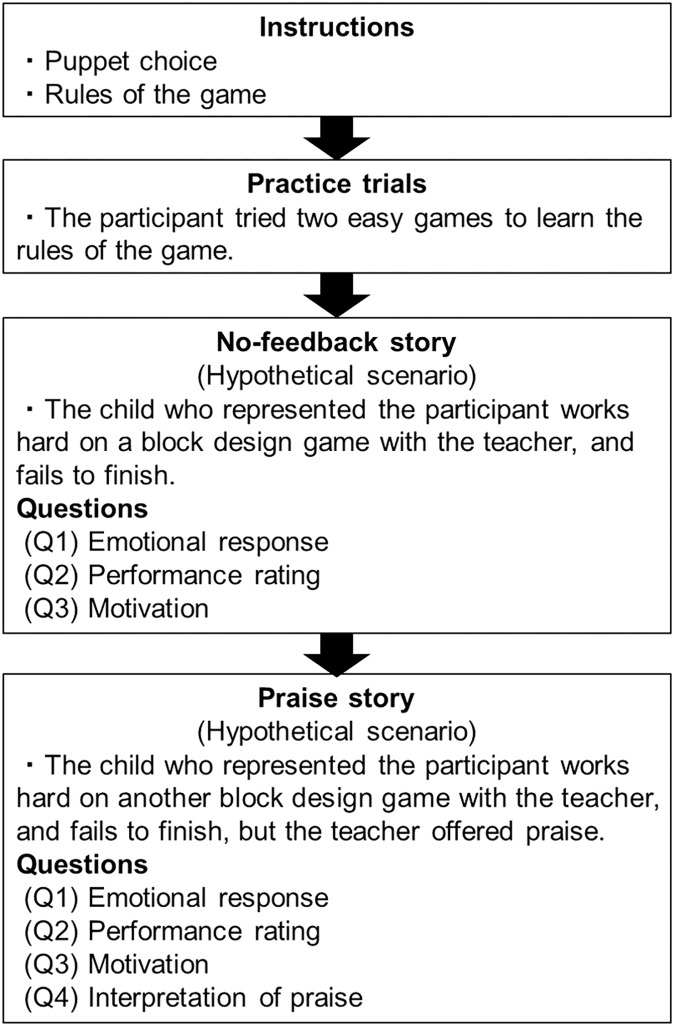
Flow of the procedure for the failure-praise task.

In the failure-praise task, the experimenter read two puppet-based stories (a “no-feedback story” and a “praise story”) aloud and directed the children to act out the role of the main character simultaneously, using a puppet. The task flow is shown in **Figure 1**. Before the experimenter read the stories, the children were instructed to choose one of four puppets to represent themselves and introduced it to another puppet that represented the teacher. The children were told that the puppet child (representing the participant) and the puppet teacher were going to play a block design game that involved the use of four bicolored blocks from the Wechsler Preschool and Primary Scale of Intelligence Block Design test (Wechsler, 2002). In the block design game, the puppet teacher showed the puppet child a design on a picture card. The puppet child was asked to recreate the design using the blocks, within a limited amount of time.

To ensure that the children understood the rules of the game, they were provided with similar, but easier, examples and allowed to practice. Upon completion of the two practice trials, they were told the no-feedback story (control story) first, followed by the praise story (test story). This was to avoid the effect of praise on the response in the no-feedback story (control story) and to confirm how they evaluate their failure itself. The two stories contained descriptions of very similar events. In both stories, the child failed to recreate a design, which was shown on a picture design card, within a limited amount of time. The difference between the two stories was that praise was provided in the praise story but not the no-feedback story. The no-feedback story was used as the control story, mainly to confirm whether children were dissatisfied with their performance when they failed.

In the no-feedback story, the children were told that the main character (i.e., the child) played a block design game and tried hard to recreate a complex design with the blocks but failed to do so within the time limit. In the praise story, the main character played another block design game with a similar degree of difficulty and tried hard to recreate the design but failed to do so. However, in the praise story, the teacher offered the main character insincere praise without a reason (i.e., “*sugoi-ne!*” in Japanese that means “great!” in English), in response to the work. The two picture design cards used in the stories were counterbalanced.

The following three questions were posed after each of the stories: (Q1) Emotional response: “I want to know how you feel about what happened in this block design game” followed by, “Do you feel happy or not? Do you feel sad or not? Do you feel angry or not?” They answered each question after being shown illustrations of each emotional facial expression. To create an index of a general positive emotional response, one point was awarded for each positive emotion (i.e., happy, not sad, and not angry), and a summed score was calculated (scores ranged from 0 to 3). (Q2) Performance rating: “Think again about everything that happened in this block design game. Should you get a circle (good) or a cross (not good) for what you did?” Children were asked to assign a grade to their block design by pointing to a circle (which means “good” in Japan) or a cross. One point was awarded for a positive evaluation (good), and no points were awarded for a negative evaluation (not good). (Q3) Motivation: “Think again about everything that happened with this block design game. If the teacher asked you to try the game again, would you like to play the difficult game or the easier game?” The children were asked this question to measure their motivation for attempting to perform challenging activities in the stories. They were asked to choose between a card depicting a complex design and a card depicting a straightforward design. One point was awarded for choice of the challenging activity, and no points were awarded for choice of the easy activity.

The children were asked another question about interpretation of praise in the praise story, as the praise provided in the story was ambiguous, and they could have interpreted it in diverse ways: (Q4) Interpretation of praise: “Why did the teacher say, ‘great!’ (*sugoi-ne!*)?” Children’s responses were classified into three categories: “outcome,” “effort,” and “other” (i.e., other interpretation, do not know, and no response). If the children did not answer, they were asked the following forced-choice question: “Did she offer the praise because you did good work (outcome) or because you tried hard (effort)?” Six of the 72 children (8.33%) were asked the forced-choice question. The author and a second coder coded participants’ interpretation of the praise. Interrater agreement, established using Cohen’s kappa, was high (*K* = 0.85). Disagreements were resolved via discussion. In the debriefing, the children and the experimenter played a block design game without a puppet, if the children wanted to try to recreate the design themselves, and they talked about successful experiences in their daily lives.

#### Theory of Mind Tasks

The children completed theory of mind tasks that included two first-order false-belief tasks (Harris et al., 1989) and two second-order false-belief tasks (Sullivan et al., 1994). The example of stories and questions used in the theory of mind tasks are shown in **Appendix 2**. These tasks have demonstrated good test-related reliability (Hughes et al., 2000). Each of the first-order false-belief tasks included two memory control questions, a first-order false-belief question, and a reality question. If the children answered all four questions correctly, they received one point. Each of the second-order false-belief tasks included a first-order false-belief question, a reality question, a second-order false-belief question, and two memory control questions. The children answered the second-order false-belief question and memory control questions only if they had answered the first two questions (the first-order false-belief and reality questions) correctly. If children answered both the first-order and reality questions correctly, they received one point for understanding first-order false beliefs, and if they answered the second-order false-belief and memory control questions correctly, they received one additional point for understanding second-order false beliefs. Therefore, overall theory of mind scores ranged from 0 to 6 (i.e., four points for understanding first-order false beliefs and two points for understanding second-order false beliefs).

#### Verbal Ability Test

Children’s vocabulary was assessed to control for verbal ability in the analysis, as this ability is typically positively related to their performance in theory of mind tasks (Milligan et al., 2007). The Picture Vocabulary Test-Revised (PVT-R; Ueno et al., 2008), which was standardized for use with Japanese samples, was used to measure children’s receptive vocabulary. The PVT-R is the Japanese version of the Peabody Picture Vocabulary Test (Dunn and Dunn, 2007). In the task, children were shown an array of four pictures on each page of a stimulus book and asked to select an appropriate picture, which was named by the experimenter, from the four pictures. The standardized score was used for the following analyses.

## Results

Data from 52 children (18 boys and 34 girls) who evaluated their work in the no-feedback story as “not good” (low performance rating after failure without feedback) were included in the main analysis examining their responses to the praise in the praise story. This data selection process was essential because young children tend to be unrealistically overconfident of their ability (e.g., Schneider, 1998) and could be unable to recognize their failure in the story. **Table 1** shows the means and standard deviations for data regarding age, PVT-R scores, theory of mind scores, and scores for each measure in the failure-praise tasks, for the 52 children.

**Table 1:** Descriptive statistics.

Measure	*M* (*SD*)	Range
Age in months	68.67 (7.42)	55–81
Verbal ability	26.31 (10.52)	6–46
Theory of mind	3.38 (1.92)	0–6
**No-feedback story**		
Emotional response	0.92 (0.65)	0–3
Performance rating	0.00 (0.00)	0
Motivation	0.73 (0.45)	0–1
**Praise story**		
Emotional response	2.33 (0.96)	0–3
Performance rating	0.58 (0.50)	0–1
Motivation	0.58 (0.50)	0–1

*Note. n = 52.*

Concerning an overview of the analysis, first, children’s responses to the praise story were compared with their responses to the no-feedback story by using Wilcoxon signed-rank tests to test the first hypothesis. To test the second hypothesis, their interpretation of the praise following failure was coded. Lastly, a logistic regression analysis concerning their responses to praise following failure per their theory of mind development was conducted to test the third hypothesis.

### Children’s Response to Insincere Praise

To test the first hypothesis, which expected that young children would show a negative response to insincere praise, Wilcoxon signed-rank tests for positive emotion, self-rated performance, and motivation were performed. Children’s levels of positive emotion (*Z* = −5.53, *p* < 0.001), and self-rated performance (*Z* = −5.48, *p* < 0.001) were significantly higher relative to those observed in the no-feedback story. Their motivation was significantly lower relative to those observed in the no-feedback story (*Z* = −2.53, *p* = 0.01).

### Children’s Interpretation of Insincere Praise

To test the second hypothesis, which predicted that most participants would attribute the receipt of insincere and ambiguous praise following failure to their own effort in a story, children’s interpretation of the praise was coded. The interpretation provided by seven of the 52 children was categorized as “other,” and the remaining 45 children were classified into one of two groups based on their attribution of the praise: 27 were classified into the outcome-interpretation group (e.g., children who believed that the teacher had offered praise because the recreated puzzle had been good or they had been able to recreate half of the puzzle) and 18 were classified into the effort-interpretation group (e.g., children who believed that the teacher had offered praise because they had worked hard or done their best to complete the challenging puzzle). Age, *t*(43) = −0.74, *ns*; PVT-R scores, *t*(43) = 1.24, *ns*; and theory of mind scores *t*(43) = 0.13, *ns* did not differ significantly between outcome- and effort-interpretation groups.

### Theory of Mind and Responses to Insincere Praise

A logistic regression analysis was performed to test the third hypothesis, which predicted that children with mature theory of mind would respond more negatively to the praise following failure, relative to children with less mature theory of mind. Coding of the interpretation of insincere praise following failure revealed that there were two major types of interpretation (i.e., effort and outcome)^1^. Thus, whether the interaction between theory of mind and the interpretation were significantly associated with their response to insincere praise was also analyzed. First, theory of mind scores were centered, and interpretation of praise was coded into effort (+1) and outcome (−1). To conduct the logistic regression analysis, children’s scores for the emotional response question were divided into two categories reflecting higher (2 and 3 points) and lower (0 and 1 points) levels of positive emotion. As dependent variables, children’s responses to praise (emotional response, performance rating, and motivation) were examined in each separate analysis. In each analysis, age and PVT-R scores were entered in the first step, theory of mind scores and interpretation of the praise were entered in the second step, and the interaction terms of theory of mind scores and interpretation of the praise were entered in the final step.

**Table 2** shows the results of the logistic regression analysis for the three dependent variables. For emotional response, the model was not significant, *χ*^2^(5) = 5.00, *p* = 0.42. For performance rating, the model was significant, *χ*^2^(5) = 14.56, *p* = 0.01, and the main effect of theory of mind was significant, *B* = −0.87, Wald = 6.90, *p* = 0.01, indicating that mature theory of mind was related to relatively lower performance ratings after insincere praise. Although the model for motivation did not reach significance, *χ*^2^(5) = 9.11, *p* = 0.11, the interaction between theory of mind and interpretation of the praise approached significance, *B* = −0.38, Wald = 3.80, *p* = 0.05. A simple slope analysis revealed that theory of mind score was negatively associated with motivation after praise following failure in the effort-interpretation group, *B* = −0.60, Wald = 3.11, *p* = 0.08, indicating that there was a tendency that children with mature theory of mind displayed lower motivation after insincere praise, relative to children with less mature theory of mind in this group. On the other hand, for children in the outcome-interpretation group, theory of mind was not significantly related to motivation, *B* = 0.15, Wald = 0.22, *p* = 0.64.

**Table 2:** Results of the logistic regression analysis (final model).

Variable	*B*	*SE*	Wald	*df*	Odds ratio	%ΔOdds	95% *CI*
**Emotional response**							
Age	0.05	0.08	0.37	1	1.05	+5	0.90–1.23
Verbal ability	0.07	0.06	1.40	1	1.08	+8	0.95–1.22
ToM	−0.10	0.29	0.11	1	0.91	−9	0.51–1.61
Interpretation (effort, outcome)	−0.04	0.44	0.01	1	0.96	−4	0.41–2.26
ToM × Interpretation	−0.07	0.23	0.10	1	0.93	−7	0.60–1.45
**Performance rating**							
Age	−0.12	0.09	1.86	1	0.89	−11	0.75–1.05
Verbal ability	0.17	0.07	6.68^∗∗^	1	1.19	+19	1.04–1.35
ToM	−0.87	0.33	6.90^∗∗^	1	0.42	−58	0.22–0.80
Interpretation (effort, outcome)	0.50	0.44	1.28	1	1.64	+64	0.70–3.88
ToM × Interpretation	−0.26	0.24	1.24	1	0.77	−23	0.49–1.22
**Motivation**							
Age	0.07	0.07	1.05	1	1.08	+8	0.94–1.24
Verbal ability	0.05	0.05	0.89	1	1.05	+5	0.95–1.16
ToM	−0.23	0.27	0.74	1	0.80	−20	0.47–1.34
Interpretation (effort, outcome)	−0.33	0.37	0.82	1	0.72	−28	0.35–1.47
ToM × Interpretation	−0.38	0.20	3.80^†^	1	0.68	−32	0.47–1.00

*Note 1. n = 45, ^†^p < 0.10, ^∗^p < 0.05; SE, standard error; CI, confidence interval; ToM, Theory of mind. Note 2. Emotional response: model fit, χ^2^(5) = 5.00, p = 0.42, -2 Log likelihood = 40.04, Nagelkerke R^2^ = 0.17, goodness-of-fit test (Hosmer–Lemeshow); χ^2^(7) = 9.88, p = 0.20. Note 3. Performance rating: model fit, χ^2^(5) = 14.56, p = 0.01, -2 Log likelihood = 46.01, Nagelkerke R^2^ = 0.37, goodness-of-fit test (Hosmer–Lemeshow); χ^2^(7) = 6.13, p = 0.53. Note 4. Motivation: model fit, χ^2^(5) = 9.11, p = 0.11, -2 Log likelihood = 52.18, Nagelkerke R^2^ = 0.25, goodness-of-fit test (Hosmer–Lemeshow); χ^2^(7) = 11.96, p = 0.10.*

## Discussion

This study examined young Japanese children’s interpretations of and responses to insincere praise following failure and explored the association between these responses and the maturity of their theory of mind. Children heard stories involving the receipt of insincere praise from a teacher following failure and answered questions regarding their interpretation of the praise, emotion, performance rating, and motivation.

Regarding children’s responses to the praise, the results showed that the children were generally happy to receive insincere praise following failure. Inconsistent with the first hypothesis, they exhibited positive emotions and their self-rated performance levels increased after the praise. The findings indicate that some of the children increased the positivity of their own evaluations to match those of the teacher. However, motivation to accept the challenge of completing a more demanding task was lower in the situation involving failure followed by praise relative to that observed in the situation involving failure without feedback, suggesting that the receipt of insincere praise from a teacher following failure could negatively affect young children’s motivation. This finding suggests that young children began to notice insincerity in praise in failure situations to some extent, but they were still affected by its positive face value.

Regarding children’s interpretation of insincere praise without an apparent reason (i.e., “great!” or “*sugoi-ne!*” in Japanese), the results did not support the second hypothesis, which predicted that most of the children would attribute the praise to their effort rather than outcomes or their personal attributes. Approximately 52% of the children attributed the praise to outcome, and approximately 34% attributed the praise to their effort. It should be noted that theory of mind scores did not differ significantly between the outcome-interpretation and effort-interpretation groups, confirming that the difference in interpretation did not reflect children’s ability to understand others’ mind; rather, it may reflect one’s attribution tendencies. Although there are no empirical data to explain the reason that about half of the children attributed the praise to outcome even in the failure situation, one interpretation was that some young children still have a tendency to interpret their work positively and with optimism; specifically, they focus on the beauty of their work rather than the components that require improvement (Lockhart et al., 2002).

The main topic of interest in this study was the association between individual differences in the maturity of children’s theory of mind and their responses to insincere praise. The results provided partial support for the third hypothesis, which predicted that children with mature theory of mind would receive and respond to the praise negatively. Children with mature theory of mind reported lower levels of self-rated performance, relative to those reported by children with less mature theory of mind after praise in both interpretation groups. One possible explanation for this finding is that children who attributed the praise to their outcome might have questioned what was in the teacher’s mind in a failure situation; for example, “She praised me; however, did she really think I gave a great performance?,” and this could have led them to give themselves a low performance rating. Children who attributed the praise to their effort might have interpreted that the teacher had no choice but to give praise for their effort because their performance was unsatisfactory, and this could have led them to give themselves a low performance rating.

In the effort-interpretation group, children with mature theory of mind also exhibited lower levels of motivation for perseverance relative to those observed for children with less mature theory of mind, following praise. They might have doubted the teacher’s praise; e.g., “She praised me; however, did she really think I tried well?” and might have answered, “Yes, I trust her. She thinks I try hard even if I have failed; therefore, I cannot be expected to do more than this.” This kind of thought might have decreased their motivation to perform more challenging tasks.

The findings revealed that children’s interpretation of insincere praise differed depending on the maturity of their theory of mind. Specifically, although theory of mind did not account for the children’s positive emotional responses, it explained the observation of lower levels of self-rated performance following the praise in those with mature theory of mind in both groups. Furthermore, theory of mind tended to be associated with weaker motivation for meeting the challenge of the task following the praise in the effort-interpretation group. It could be that young children with mature theory of mind recognize the uncertainty in the teacher’s insincere praise and therefore considered the feedback as unreliable. One could also consider the idea that some children interpreted “great (*sugoi-ne*)” as a sarcastic remark. However, it is unlikely that the participants interpreted the praise as sarcasm. This is because most children attributed the praise to their effort or outcome and did not reported sarcastic intentions, and most of them exhibited positive emotion following the praise. The remark would be more likely be interpreted as sarcastic at the age of approximately 9 years, when children understand irony (Filippova and Astington, 2008; Massaro et al., 2013).

Most previous studies examining children’s responses to praise have focused on positive feedback following success (Mueller and Dweck, 1998; Kamins and Dweck, 1999; Henderlong and Lepper, 2002; see also Brummelman et al., 2014). Within this body of literature, the main strength of this study is its novel focus on insincere praise following failure, and the new finding that process-related praise, which was believed to have positive effect on children’s self-evaluation and motivation (cf. Kamins and Dweck, 1999), has a negative effect in some cases. This implies that praise for effort in a failure situation sometimes conveys the message that adults are sufficiently satisfied with children’s effort, thus decreasing children’s motivation for challenges. The evidence of the current study indicated that the effects of insincere praise could differ depending on the maturity of children’s theory of mind. The findings also have practical implications that there is a need for fine-grained observation of individual children’s understanding of mental states prior to the provision of evaluative feedback. As theory of mind dramatically develops in young children, parents and teachers should avoid using thoughtless positive feedback and assess how children evaluate their own work.

This study was the first to perform an empirical examination of insincere praise and revealed the association between young children’s negative responses to the praise following failure and the development of theory of mind. Although the study demonstrated strengths and important implications, it was subject to some limitations. For example, children’s responses to the praise following failure were examined within the context of the scenarios, and the ways that children respond to this type of praise in their everyday lives remains unclear. Another limitation involves the nature of the sample, which included a relatively small sample size and an unbalanced sex distribution. It should also be noted that Japanese children could be motivated by failure, as they have been shown to work harder following failure than they do following success (Heine et al., 2001). Additional studies specifically designed to examine the effects of praise in diverse settings and cultures are required to provide evidence for or against the generalization of the current findings.

## Author Contributions

The author confirms being the sole contributor of this work and approved it for publication.

## Conflict of Interest Statement

The author declares that the research was conducted in the absence of any commercial or financial relationships that could be construed as a potential conflict of interest.

## Footnotes

1 Data from seven children whose interpretation was classified as “other” were eliminated from analyses.

## Appendix 1. Stories Used in the Failure-Praise Task

### Instruction

You are playing a block design game with *Hanako-sensei* (teacher Hanako). *Hanako-sensei* shows you four blocks; one side of these blocks has two colors—white and red—and the other side is colored red.

### No-Feedback Story

*Hanako-sensei* shows you a picture of a model and says, “Look at this picture and recreate the design yourself using these blocks. Start!” You try hard to recreate the design but fail to do so.

### Praise Story

*Hanako-sensei* shows you a picture of another model and says, “Look at this picture and recreate the design yourself using these blocks. Start!” You try hard to recreate the design but fail to do so. *Hanako-sensei* comes to your side and says, “[Child’s name], *sugoi-ne* (great)!”

## Appendix 2. Example of Stories and Questions Used in the Theory of Mind Tasks

### First-Order False-Belief Task

I’m going to tell you a little story about *Saru-san* (Monty) and his lunch box. Look! Here’s *Saru-san*. He wants *Kaeru-san* (Freddie) to put an apple in his lunch box to take to school, but *Kaeru-san* says there are no apples left, so he’ll have to take a pear instead. *Saru-san* doesn’t like pears at all. He really wanted an apple! He’s so cross about the pear that he stamps his feet all the way up the stairs.

Q1. How does Saru-san feel when he gets a pear? (Memory control 1)
Q2. How does Saru-san feel when he gets an apple? (Memory control 2)

But, look! While *Saru-san* is out of the kitchen, *Kaeru-san* finds one apple left in the cupboard. He decides to give *Saru-san* a nice surprise; so, he takes the pear out of *Saru-san*’s lunchbox and puts the apple in there instead. He puts the lunchbox in *Saru-san*’s bag. *Saru-san* comes back, picks up the bag, and hurries off to school. *Saru-san* doesn’t see what’s inside his lunchbox. Now it’s lunchtime. *Saru-san* takes out his lunchbox.

Q3. What does Saru-san think is in the box, an apple or a pear? (First-order false-belief)
Q4. What is really in the box, an apple or a pear? (Reality)

### Second-Order False-Belief Task

This is Kouta. Today is his birthday, and Kouta’s mom is going to surprise him by giving him a puppy. She has hidden the puppy in the shed until it’s time for Kouta’s birthday party. Kouta says “I really hope you’ve got me a puppy for my birthday, Mom”; but, remember, Mom wants to surprise Kouta; so, instead of telling Kouta she got him a puppy, Mom says, “Sorry, I didn’t get you a puppy, Kouta. Actually, I’ve got you a really good toy for your birthday.”

Q1. What did Kouta think he was getting for his birthday? (First-order false-belief)
Q2. What was his mom really giving him? (Reality)

[If the child answers the two questions correctly, continue with the following story].

Now Kouta decides to go outside to play. On his way out, he goes into the shed to get his bike, and he finds the birthday puppy! Kouta says to himself, “Wow! Mom didn’t get me a toy; she really got me a puppy for my birthday!” Mom didn’t see Kouta go to the shed; so, she doesn’t know he found the birthday puppy. Inside, the telephone rings. It’s Kouta’s granny, calling to find out what time the party is. Granny says to Mom, “What does Kouta think you’ve gotten him for his birthday?”

Q3. What does Mom say to Granny? (Second-order false-belief)
Q4. Did Mom see Kouta go into the shed? (Memory control 1)
Q5. What did Mom really get Kouta for his birthday? (Memory control 2)
